# Utilizing chemically induced dimerization of FKBP to analyze endocytosis by live-cell imaging in budding yeast

**DOI:** 10.1016/j.xpro.2022.101323

**Published:** 2022-04-14

**Authors:** Andrew K. Lamb, Santiago M. Di Pietro

**Affiliations:** 1 Department of Biochemistry and Molecular Biology, Colorado State University, Fort Collins, CO 80523-1870, USA

**Keywords:** Cell Biology, Cell-based Assays, Microscopy, Model Organisms, Signal Transduction

## Abstract

Chemically induced dimerization (CID) is a useful tool for artificially inducing protein-protein interactions. Although CID has been used extensively for live-cell microscopy applications in mammalian systems, it is rarely utilized in yeast cell biology studies. Here, we present a step-by-step protocol for the utilization of a CID system in live-cell microscopy experiments of budding yeast endocytosis. While focusing on the study of endocytosis, this protocol framework is adaptable to the study of other cellular processes in *Saccharomyces cerevisiae*.

For complete details on the use and execution of this protocol, please refer to Lamb et al. (2021).

## Highlights

• Generation of yeast strains for endogenous expression of FKBP-tagged proteins
• Utilization of an inducible homodimerization system in *S. cerevisiae*
• Fluorescence microscopy imaging of clathrin-mediated endocytosis

## Graphical abstract

**Figure:**
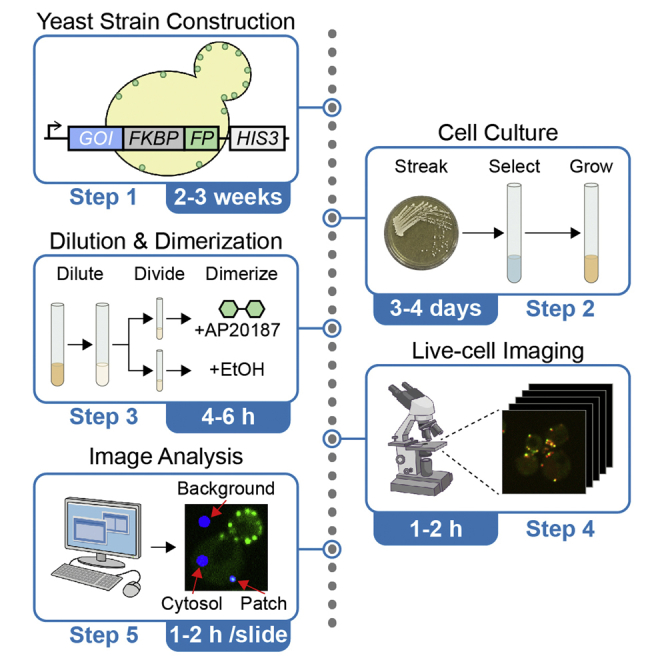

## Before you begin

**Figure 1:**
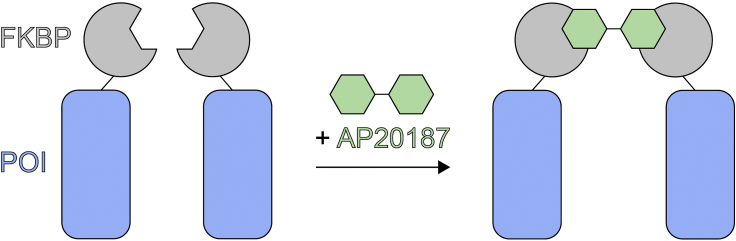
AP20187 induces homodimerization of the FKBP domain

Protein-protein interactions are critical for the subcellular localization and activity of proteins in numerous cellular processes. Advances in chemically induced dimerization (CID) systems have allowed researchers to artificially induce protein-protein interactions (Clackson, 1998). CID systems rely on small cell-permeable chemicals that bind to a pair of proteins with high affinity. At the forefront of CID systems is the FK506-binding protein (FKBP) family, whose members are low molecular weight proteins that are present in all eukaryotes (Kolos et al., 2018). Through the work of countless researchers, a diverse set of FKBP ligands have been derived, allowing for various chemically inducible processes (Voß et al., 2015). One such ligand is AP20187, a chemically engineered bivalent ligand that binds two FKBP molecules. By fusing FKBP to a protein of interest, treatment with AP20187 allows for homodimerization of two chimeric proteins (Figure 1). This inducible homodimerization system allows for the study of proteins that require oligomerization for their proper localization or activity, among other applications (DeRose et al., 2013).

Clathrin-mediated endocytosis (CME) is an essential endocytic pathway that is well conserved from yeast to humans (McMahon and Boucrot, 2011; Weinberg and Drubin, 2012). Given this and the tractability of *S. cerevisiae* genetics, budding yeast has become a prominent model for the study of CME (Wendland et al., 1999; Newpher et al., 2005; Kaksonen et al., 2005). In yeast, CME involves about 60 endocytic proteins which are sequentially recruited to endocytic patches along the plasma membrane (Weinberg and Drubin, 2012; Boettner et al., 2012; Goode et al., 2015). These proteins work to invaginate the surrounding membrane and pinch the invagination off as a vesicle inside the cell. CME proteins have been studied extensively through the use of live-cell fluorescence microscopy, which allows researchers to visualize the spatial and temporal dynamics of proteins at endocytic patches (Kaksonen et al., 2005; Toshima et al., 2006; Mettlen and Danuser, 2014). As is the case with other cellular processes, the recruitment and activity of endocytic proteins are highly dependent on a complex network of protein-protein interactions.

Here, we present a procedure for utilizing a chemically induced dimerization system on endocytic proteins and visualizing the effects of homodimerization through live-cell imaging. While this protocol is tailored for the imaging and analysis of proteins involved in CME, the application of the CID system can be utilized for the study of other cellular processes.

### Yeast strain construction

**Timing: 2–3 weeks**

**Figure 2:**
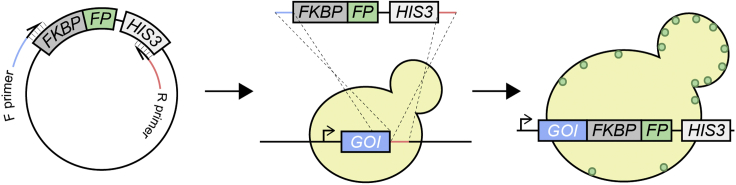
Schematic of C-terminal tagging of gene of interest (GOI) with FKBP and fluorescent protein (FP)

Prior to the experiment, a yeast strain must be engineered to endogenously express a protein of interest tagged with both FKBP and a fluorescent protein (FP). These tags will allow for induction of homodimerization and the subsequent monitoring of the protein through live-cell fluorescence microscopy, respectively. In addition, it may be desirable to include additional FP reporters or gene deletions in the strain(s). Below, we discuss the steps of a potential strategy for construction of yeast strains (Figure 2). This single step PCR-based method will allow for C-terminal tagging of the protein of interest (Longtine et al., 1998). Detailed step-by-step protocols for this process have been outlined elsewhere (Gardner and Jaspersen, 2014). If N-terminal tagging is required, we suggest using an alternative 2-step strategy to avoid disruption of the native promoter (Gardner and Jaspersen, 2014).

1. **Construct a plasmid containing FKBP in frame with an FP, followed by a selectable marker:** Numerous plasmids, such as pFA6A derivatives, are available commercially that encode an FP upstream of a selectable marker which allows for simple PCR-based tagging of genes. These plasmids often contain multiple cloning sites upstream of the FP. Using standard molecular cloning techniques, introduce the sequence encoding FKBP upstream and in-frame with the FP. Alternatively, we have generated the plasmid pFA6a-FKBP-GFP-HIS3MX6 (Addgene #183023), which encodes FKBP upstream of GFP and the HIS3MX6 selectable marker.

***Note:*** Ensure that the FKBP sequence encodes for the F36V mutant of FKBP. Chemically engineered ligands such as AP20187 have increased specificity for FKBP^F36V^ over wild-type FKBP (Clackson et al., 1998).

2. **Amplify the tagging cassette via PCR and transform the product into yeast:** Design a pair of primers that amplify the entire tagging cassette (FKBP-FP-Selection Marker). In addition, the primers should include ∼40 additional nucleotides at their 5′ ends that are homologous to the sequences directly upstream (F Primer) and downstream (R Primer) of the gene of interest stop codon. Ensure that the selection marker is compatible with the yeast strain utilized. We commonly use BY4741 as a background yeast strain (Brachmann et al., 1998) and introduce the PCR product into yeast cells by lithium acetate transformation (Ito et al., 1983).
3. **Verify integration by colony-PCR and ensure functionality of the fusion protein:** Using colony-PCR, verify that the PCR product was integrated at the correct locus. Sequence the colony-PCR product to verify that no mutations were introduced during the procedure. To ensure the FKBP tag does not affect protein expression or localization, a strain should be generated where the protein of interest is tagged with only the FP for comparison. Compare the expression levels of the fusion proteins from the two strains through western blotting of total cell extract, and check localization of the proteins through live-cell imaging (Feliciano et al., 2011). Additionally, ensure the tags do not affect yeast growth by comparing the growth rates of the tagged strains to the untagged background strain (Pringle and Mor, 1975).
4. **Incorporate additional FP reporters or gene deletions:** It may be desirable to include additional fluorescent-labeled proteins or gene deletions within the strain. This can be accomplished through mating the FKBP strain with another strain containing the desired allele (Lichten, 2014). Alternatively, additional FP tags or deletions can be introduced through subsequent steps of PCR-based homologous recombination.

## Key resources table

**Table:** 

REAGENT or RESOURCE	SOURCE	IDENTIFIER
**Chemicals, peptides, and recombinant proteins**		

Adenine	Sigma-Aldrich	Cat# A9795
AP20187 (B/B Homodimerizer)	TAKARA BIO	Cat# 635059; CAS 195514-80-8
Bacto Agar	BD Biosciences	Cat# 212750
Bacto Peptone	BD Biosciences	Cat# 211677
Bacto Yeast Extract	BD Biosciences	Cat# 212750
Dextrose (D-glucose)	Fisher Scientific	Cat# D16
Difco Yeast Nitrogen Base (YNB) w/o Amino Acids	BD Biosciences	Cat# 291920
Ethanol, 200 Proof	PHARMCO-AAPER	Cat# 111000200
L-Methionine	Sigma-Aldrich	Cat# M5308
L-Histidine	Sigma-Aldrich	Cat# H8125
L-Leucine	Sigma-Aldrich	Cat# L8000
L-Tryptophan	Sigma-Aldrich	Cat# T0254
L-Lysine	Sigma-Aldrich	Cat# L5626
Uracil	Sigma-Aldrich	Cat# U0750

**Experimental models: Organisms/strains**		

*S. cerevisiae* strain SDY1377: *MATa his3Δ1, leu2Δ0, met15-Δ0, ura3Δ0, ABP1-mCherry::HIS3, AIM21-FKBP-GFP*	Lamb et al. (2021)	N/A
*S. cerevisiae* strain SDY1378: *MATa his3Δ1, leu2Δ0, met15-Δ0, ura3Δ0, ABP1-mCherry::HIS3, AIM21-FKBP-GFP, tda2Δ::URA3*	Lamb et al. (2021)	N/A

**Recombinant DNA**		

pFA6a-FKBP-GFP-HIS3MX6	This paper	Addgene Plasmid #183023

**Software and algorithms**		

GraphPad Prism 9	GraphPad Software	https://www.graphpad.com/
SlideBook 6	Intelligent Imaging Innovations	https://www.intelligent-imaging.com/slidebook

**Other**		

Incubator	Fisher Scientific	Cat# 11-690-650D
Culture tube rotator	Glas-Col	Cat# 099A-RD4512
Culture tubes (14 mL)	Fisher Scientific	Cat# 14-956-6B
Culture tubes (20 mL)	Sigma-Aldrich	Cat# CLS982016XX
Cell density meter	Amersham	Cat# 80-2116-31
Disposable cuvettes	Fisher Scientific	Cat# 14955128
1.5 mL Microcentrifuge tubes	Labcon	Cat# 3016-870-000-9
Microcentrifuge	Eppendorf	Cat# 5401000137
Microcopy slides	Fisher Scientific	Cat# 12-544-3
Microscopy coverslips (22 mm × 22 mm)	Fisher Scientific	Cat# 12-541-B
Objective Immersion Oil	Olympus	Cat# NC0297589
Microscopy System: IX81 inverted spinning-disk confocal microscope (Olympus) with an Andor iXon Ultra camera (Oxford Instruments) and a 100× 1.40 NA objective. Excitation was performed with diode lasers of 488 nm (GFP) and 561 nm (mCherry). The microscope has a CSU 22 head with quad dichroic and additional emission filter wheels to eliminate spectral crossover. The emission filters used were 521 ± 12.5 nm and 607 ± 12.5 nm.	N/A	N/A

## Materials and equipment

**Table:** 50% Dextrose

Reagent	Final concentration	Amount
Dextrose	50% (w/v)	250 g
ddH_2_O		Up to 500 mL
**Total**		**500 mL**

Heat at 50°C on a stir plate to dissolve. Sterilize by autoclaving or filtration. Store at 20°C–22°C for up to 1 year.

**Table:** Yeast Extract-Peptone-Dextrose (YPD) Plates

Reagent	Final concentration	Amount
Bacto Yeast Extract	2% (w/v)	14 g
Bacto Peptone	1% (w/v)	7 g
Bacto Agar	2% (w/v)	14 g
∗50% Dextrose∗	2% (w/v)	28 mL
ddH_2_O		670 mL
**Total**		**∼700 mL**

Autoclave the mixture without ∗dextrose∗ in a flask with a magnetic stir bar. Following the autoclave cycle, add dextrose. Stir solution gently at room temperature until solution reaches 55°C. Pour 25 mL per plate (approximately 25 plates). Leave plates overnight at room temperature, then store upside down and sealed at 4°C for up to 2 months.

**Table:** Synthetic Defined (SD) Medium

Reagent	Final concentration	Amount
Difco Yeast Nitrogen Base (YNB) w/o Amino Acids	6.7 g/L	6.7 g
50% Dextrose	2% (w/v)	40 mL
ddH_2_O		Up to 990 mL
**Total**		**990 mL**

Autoclave or filter sterilize, and store at 20°C–22°C for up to 1 year.

**Table:** 100× Complete Amino Acid Mix

Reagent	Final concentration	Amount
Adenine	4 mg/mL	400 mg
L-Histidine	2 mg/mL	200 mg
L-Leucine	3 mg/mL	300 mg
L-Lysine	3 mg/mL	300 mg
L-Methionine	2 mg/mL	200 mg
L-Tryptophan	2 mg/mL	200 mg
Uracil	2 mg/mL	200 mg
ddH_2_O		Up to 100 mL
**Total**		**100 mL**

Filter sterilize, and store at 20°C–22°C for up to 1 year in a dark location.

**Table:** Synthetic Complete (SC) Medium

Reagent	Final concentration	Amount
SD Medium	N/A	990 mL
100× Complete Amino Acid Mix	1×	10 mL
**Total**		**1,000 mL**

Mix sterilely. Store at 20°C–22°C for up to 1 year in a dark location.

## Step-by-step method details

### Yeast cell culture

**Timing: 3–4 days**

This step describes the culturing of yeast cells prior to the day of imaging.

1. Revive yeast strain(s) of interest from a glycerol stock by streaking out the strain on a YPD plate.
  a. Incubate the plate upside down at 30°C for 2–3 days.

***Note:*** YPD is a rich medium that allows for optimal yeast growth but should be avoided for liquid cultures used for microscopy due to high levels of autofluorescence.

***Note:*** Once single colonies with a 1–2 mm diameter are present, store plates upside down and covered in parafilm for up to 1 month at 4°C.

***Note:*** This step is unnecessary for newly created strains already plated and stored at 4°C.

2. The day prior to imaging, prepare a starter culture by inoculating a single colony into a culture tube containing 3 mL SC medium.
  a. Incubate in culture tube rotator at 30°C for 12–16 h.

***Note:*** Alternatively, incubation can be performed at 20°C–22°C, resulting in slightly slower growth rates. In either case, ensure the incubation takes place in a dark location and has a constant source of agitation (shaking or rotating).

***Note:*** For culture volumes under 5 mL, we commonly use disposable culture tubes with a capacity of 14 mL. For culture volumes between 5 and 10 mL, we prefer to use culture tubes with a capacity of 20 mL to prevent problems with culture aeration.

### Cell dilution and chemical dimerization

**Timing: 4–6 h**

This step describes the dilution of the starter culture, culturing cells to early logarithmic phase, and inducing chemical dimerization of the FKBP domain through addition of AP20187 to the culture.

3. The following day dilute the overnight starter culture to an OD_600_ of 0.1–0.2 in a total volume of 10 mL fresh SC medium.

**Figure 3:**
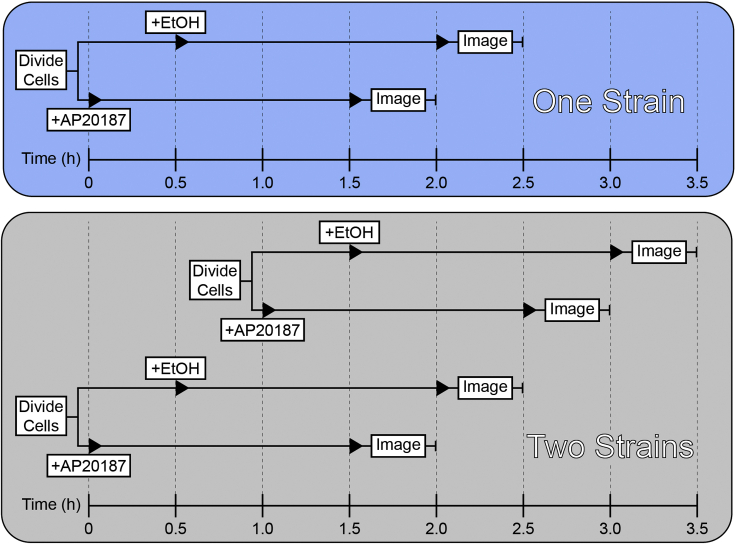
Timeline of drug treatment and live cell imaging for either one or two strains

***Note:*** 5 mL of total yeast culture will be needed for drug treatment. An initial volume of 10 mL will allow for numerous OD_600_ measurements, while not running the risk of running low on culture.

***Note:*** We typically dilute overnight cultures 20-fold in fresh SC medium (500 μL overnight culture, 9,500 μL SC medium), resulting in an OD_600_ ∼0.15.

***Note:*** If multiple strains are being imaged, staggering the dilution times by an hour will allow for minimal overlap between each step (Figure 3).

4. Incubate the diluted culture in rotator at 30°C for 3–5 h, allowing cells to grow to early logarithmic phase (OD_600_ 0.2–0.3).
5. Transfer 2.5 mL of early log phase cells to two new culture tubes labeled for drug (AP20187) or vehicle (ethanol) treatment, respectively.
  a. Return the ethanol treatment tube to 30°C incubator.

***Note:*** We purchase AP20187 as a 0.5 mM solution in 100% ethanol, however it can also be purchased as a solid chemical. If purchased as a solid, dissolve in 100% ethanol to a concentration of 0.5 mM, and store at −20°C.

**CRITICAL:** Including the vehicle (ethanol) treatment cells will be crucial for proper analysis.

6. Add 25 μL of 0.5 mM AP20187 (5 μM final) to the AP20187 treatment tube.
  a. Return to the incubator and rotate at 30°C for 90 min.

**Figure 4:**
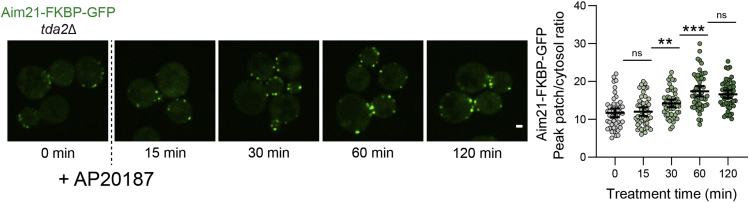
Pilot experiment for determining optimal AP20187 treatment duration Aim21-FKBP-GFP recruitment to endocytic sites increases over the 60 min following AP20187 treatment, then remains constant from 60 min to 120 min. Scale bar, 1 μM. Error bars, mean with 95% CI.

***Note:*** The concentration of ethanol following drug or vehicle treatment using the outlined volumes is less than 1% (v/v), posing no threat to the health of budding yeast cells.

***Optional:*** The optimal incubation time of cultures with AP20187 may vary depending on the protein of interest. To determine the optimal incubation time of cultures with AP20187, it may be beneficial to perform a pilot experiment with multiple drug treatment times (Figure 4).

7. 30 min after AP20187 treatment, remove the ethanol treatment tube from the incubator and add 25 μL of 100% ethanol.
  a. Return to the incubator and rotate at 30°C for 90 min.

***Note:*** Each treatment should take about 20 min to prepare slides and image. Staggering the drug and vehicle treatments by 30 min allows for ample time to image while keeping the treatment period constant at 90 min.

### Live-cell imaging

**Timing: 1–2 h**

This step outlines the processes of concentrating cultures, preparing slides, and imaging slides on a spinning-disk confocal microscope. Each slide will take about 20 min to prepare and image.

8. Following the 90 min incubation, transfer 1 mL of AP20187 treated cells to a 1.5 mL microcentrifuge tube.
  a. Centrifuge at 4,000 × *g* for 1 min.
9. Aspirate ∼970 μL of the supernatant, leaving behind ∼30 μL of liquid and the yeast cell pellet.
10. Resuspend the cells by pipetting up and down.
  a. Transfer 3 μL of the cell suspension to the center of a microscopy slide.
  b. Cover the cell suspension with a glass coverslip.

***Note:*** Debris on the slide or coverslip can cause some of the yeast cells to be improperly immobilized. Gently wipe slide and coverslip with a kimwipe immediately prior to slide preparation to remove any debris that may prevent the coverslip from lying flat along with the slide.

***Note:*** The coverslips used in this protocol have dimensions of 22 mm length × 22 mm width. If coverslips with different dimensions are used, the volume of cell suspension used for slide preparation may need to be adjusted.

11. Mount the prepared slide on a 100× oil immersion objective.

***Note:*** A spinning disk confocal microscope is our imaging system of choice given its acquisition speed and gentle imaging. However, alternate imaging systems may be utilized.

12. Using brightfield illumination, focus on a medial focal plane at the cross-section of a group of cells, and center the cells in the field of view.

***Note:*** For analysis of images, five cells will be analyzed per time-lapse image. To account for these, ensure that ∼10 budding cells are centered in the field of view.

13. Capture time-lapse images at 1 s intervals for 90 s with the following parameters:
  a. 488 nm laser line: 250 ms exposure.
  b. 561 nm laser line: 250 ms exposure.
  c. 50% laser power.

**CRITICAL:** Exposure and laser power must be optimized for your imaging system and protein(s) of interest to ensure adequate signal while minimizing photobleaching. These parameters should be determined prior to the experiment.

***Note:*** Excitation wavelength must be matched to the FP that is used. For this example, the 488 nm and 561 nm laser lines are used to excite GFP and mCherry fusion proteins, respectively.

***Note:*** A 90 s time-lapse duration is well suited for proteins involved in the late stages of endocytosis, such as the actin polymerization and scission machinery. For proteins with longer patch lifetimes, such as adaptor or coat proteins, time-lapse images will need to be collected over a longer duration and may require a longer interval between images to prevent photobleaching.

14. Capture 2–3 additional time-lapse images using identical parameters.
15. 90 min following ethanol treatment, repeat steps 8–14 with the ethanol-treated cells.

### Image analysis

**Timing: 1–2 h per slide**

This step outlines the analysis of individual endocytic patches from time-lapse images.

**Figure 5:**
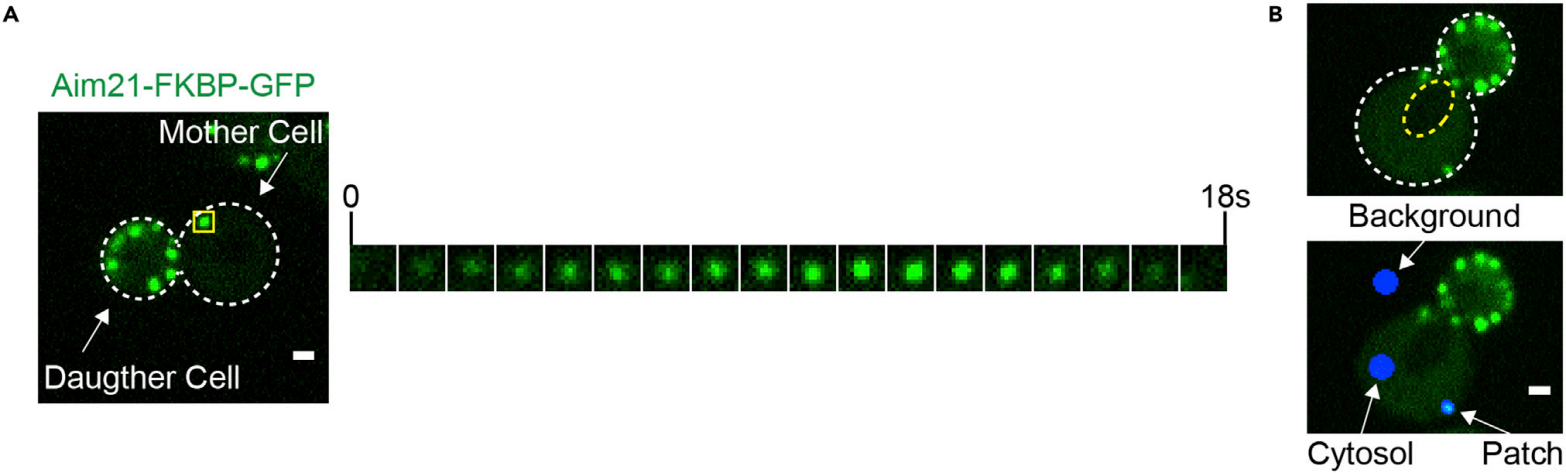
Analysis of endocytic patches from time-lapse images (A) An example live-cell microscopy image from cells expressing Aim21-FKBP-GFP. An individual endocytic patch from a mother cell (yellow box) is tracked over time in the kymograph to the right. (B) (B, top) A cell expressing Aim21-FKBP-GFP is annotated to display the cell periphery (white) and the nucleus/vacuole (yellow). (B, bottom) The background, cytosol and patch regions used for measuring fluorescence intensity during image analysis are displayed in blue. Ensure that the cytosol region does not include area from the dimmer nucleus and vacuole, if present. Scale bars, 1 μM.

***Note:*** Alternatively, image analysis can be performed using a multitude of different image analysis programs.

***Note:*** We prefer to only analyze mother cells as it is difficult to differentiate between individual endocytic patches in daughter cells, which are rich in endocytic patches (Figure 5A). Additionally, it is difficult to determine a reliable fluorescence intensity of the cytosol in patch-rich daughter cells.

16. Using SlideBook 6, open a time-lapse image and identify an actively budding mother cell to analyze. Endocytic proteins will appear in the cell as punctate structures along the cell membrane, persist for a period, then disappear (Figure 5A).

17. For the first five endocytic patches to appear within the cell, determine the following metrics:
  a. **Peak patch/cytosol fluorescence intensity ratio:** peak patch/cytosol ratio is a metric used to assess the recruitment of a protein to endocytic patches.
    i. Create a mask within the time-lapse image (select *Analyze* > *Create Mask*).
    ii. Generate regions within the mask that cover the endocytic patch of interest, the cytosol of the mother cell, and the background outside of the cell (Figure 5B). Tools for generating regions are found under *Analyze* > *Mask Generation.*
    iii. Track the patch over time and record the average fluorescence intensity values of each mask for the frame in which the average patch intensity is at its maximum.
    iv. Correct the patch and cytosol fluorescence intensities by subtracting the background fluorescence intensity.
    v. Divide the corrected patch intensity by the corrected cytosol intensity.
      $$\frac{peak\;patch}{cytosol}\;ratio=\frac{patch\;intensity_{peak}\;-\;background_{peak}}{cytosol\;intensity_{peak}\;-\;background_{peak}}$$
      ***Note:*** Ensure that the mask in the cytosolic region of the cell excludes the vacuole and nucleus (Figure 5B). Adjusting the contrast of the fluorescence image or taking a differential interference contrast (DIC) image can make identification of the vacuole and nucleus easier.
  b. **Patch lifetime:** patch lifetime is a measure of the duration that an endocytic protein is present at an endocytic patch, measured in seconds.
    i. Visually identify the starting point of the patch.
    ii. Count the number of frames in which the protein is present at the patch.
    iii. Multiply the number of frames by the time-lapse image frame interval (e.g., 15 frames × 1 s interval = 15 s patch lifetime).
      ***Note:*** Mutations that cause defects in recruitment of endocytic proteins to patches often result in an underestimation of the patch lifetime of the protein, as the protein may be present at levels below detection for much of its lifetime.

18. Repeat the analysis on 5 total cells in the time-lapse image.
19. Repeat the same analysis of 5 cells in a second time-lapse image, resulting in the analysis of 50 endocytic patches in total.
20. Complete the analysis in steps 16–20 for both AP20187 and ethanol-treated cells.
21. Compare the average peak patch/cytosol ratio and patch lifetime of AP20187 and ethanol-treated cells to determine the effect of homodimerization.

## Expected outcomes

Execution of this protocol will result in a series of time-lapse images from cells treated with AP20187 and a vehicle (ethanol) control. Through quantification of the peak patch/cytosol fluorescence intensity ratio and patch lifetime of the fluorescent-tagged endocytic proteins, the effect of oligomerization on localization and dynamics of a protein can be determined. AP20187 cells should be compared to vehicle-treated cells to assess the phenotype directly attributable to AP20187-induced homodimerization.

**Figure 6:**
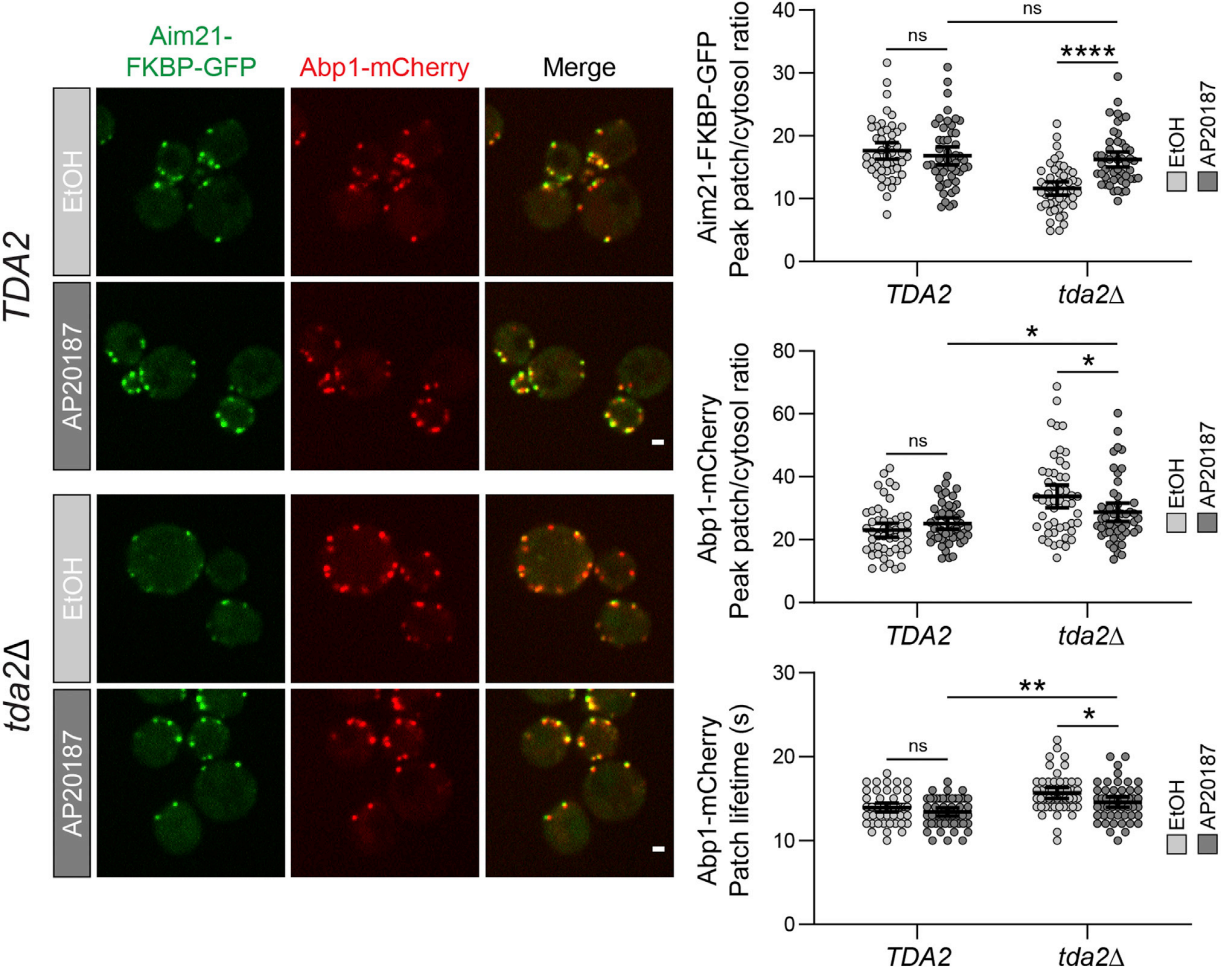
Rescue of an endocytic phenotype via AP20187-induced homodimerization Cells expressing Aim21-FKBP-GFP and Abp1-mCherry from their endogenous loci were treated with ethanol (vehicle) or AP20187 for 90 min to assess the phenotype elicited by homodimerization of Aim21-FKBP-GFP. Scale bars, 1 μM. Error bars, mean with 95% CI.

In Figure 6, an example set of experimental results is shown. Aim21 is an endocytic protein that is involved in regulation of actin polymerization during CME. Tda2-induced dimerization of Aim21 is critical for the localization and activity of Aim21. When cells lacking Tda2 (*tda2Δ*) and expressing Aim21-FKBP-GFP are treated with AP20187, the recruitment of Aim21 to endocytic sites is rescued to wild-type levels. Additionally, the levels and lifetime of the actin network at endocytic patches, as exhibited by the F-actin binding protein Abp1-mCherry, display a partial rescue.

## Limitations

This protocol utilizes the simplest form of slide preparation for yeast microscopy. While simple and effective, slides prepared this way should not be imaged for more than about 20 min. While this does not pose an issue for the study of CME, it could if one desires to image proteins involved in other cellular processes. If imaging over a longer time course is required, we suggest preparing slides with an alternative method using concanavalin A-coated coverslips (Pemberton, 2014).

To determine the efficiency of homodimerization using this protocol, a clear phenotype must be present upon treatment with AP20187. In some cases, homodimerization of a protein may have little impact on protein localization or activity. In these cases, it may be difficult to differentiate between a negative result and an experiment that is in need of optimization.

## Troubleshooting

### Problem 1

C-terminal tagging of protein of interest with FKBP affects expression, localization, or functionality of chimeric protein (before you begin: yeast strain construction).

### Potential solution

Ensure that the tag was integrated in frame with protein of interest by sequencing colony-PCR product from strain verification.
Include (or increase length of) a flexible amino acid linker between protein of interest and FKBP.
Try FKBP tag at the N-terminus instead of C-terminus. This will require a two-step process for strain construction to avoid disruption of the native promoter (Gardner and Jaspersen, 2014).

### Problem 2

Cells are not properly immobilized during live-cell imaging (step-by-step method details: live-cell imaging).

### Potential solution

Ensure that the slide and coverslip are both debris-free prior to slide preparation.
Decrease the density of the cell suspension used for live-cell imaging. Leaving ∼30 μL for resuspension of cells following centrifugation results in a suspension of the proper density.

### Problem 3

The fluorescent signal from FP is insufficient (step-by-step method details: live-cell imaging).

### Potential solution

Optimize imaging parameters (laser power and exposure time) for the protein of interest. This may require a longer interval between time-lapse images to prevent photobleaching.
Use an alternate fluorophore. We typically use GFP-based FPs as a first choice given their brightness and photostability (Bialecka-Fornal et al., 2016).
Include multiple copies of the FP (2×FP or 3×FP) in tandem.
Utilize a microscope with higher sensitivity.

### Problem 4

Treatment with AP20187 results in no clear phenotype (step-by-step method details: image analysis).

### Potential solution

Experiment with different concentrations of AP20187 used for treatment.
Experiment with different AP20187 incubation times to determine the optimal incubation time.
Tag protein of interest with FKBP at the opposite terminal, as position of dimerization may be important for biological activity.
Include multiple copies of FKBP (2× or 3×) fused to your protein of interest. This will mimic higher-order oligomerization, as opposed to simple homodimerization.

### Problem 5

High variability in peak patch/cytosol ratio and patch lifetime metrics (step-by-step method details: image analysis).

### Potential solution

Analyze all mother cells, as endocytic patches in mother and daughter cells can display different dynamics. Also analyzing mother cells containing buds of similar size (i.e., at similar cell cycle stage) can be helpful (Pedersen et al., 2020).
Ensure that the cells have re-entered the logarithmic growth phase prior to imaging. Cells that have not exited the lag phase of growth may display slower dynamics.
Ensure that the regions created for fluorescence intensity measurements properly cover the regions of interest (Figure 5B).
Proteins expressed at low levels may produce a low fluorescence intensity compared to background, resulting in high variability in metrics. See problem 3 for potential solutions.

## Resource availability

### Lead contact

Further information and requests for resources and reagents should be directed to and will be fulfilled by the lead contact, Santiago Di Pietro (santiago.dipietro@colostate.edu).

### Materials availability

Strains utilized in this study are available upon request.

## Data Availability

The example dataset generated in this study is available upon request.
